# Evaluating the benefits of a youth mental health curriculum for students in Nicaragua: a parallel-group, controlled pilot investigation

**DOI:** 10.1017/gmh.2017.27

**Published:** 2018-01-25

**Authors:** Arun V. Ravindran, Andres Herrera, Tricia L. da Silva, Joanna Henderson, Magda Esther Castrillo, Stan Kutcher

**Affiliations:** 1 University of Toronto and Centre for Addiction and Mental Health, Toronto, Ontario, Canada; 2 Universidad Nacional Autonoma de Nicaragua Leon and Centro de Investigación en Demografía y Salud (National Autonomous University of Nicaragua Leon and Centre for Demographic and Health Research), Leon, Nicaragua; 3 IWK Health Centre and Dalhousie University, Halifax, Nova Scotia, Canada

**Keywords:** Functioning, low-and-middle-income countries, mental health knowledge, school-based mental health literacy programs, stigma, youth mental health

## Abstract

**Background.:**

High rates of mental illness and addictions are well documented among youth in Nicaragua. Limited mental health services, poor mental health knowledge and stigma reduce help-seeking. The Mental Health Curriculum (MHC) is a Canadian school-based program that has shown a positive impact on such contributing factors. This pilot project evaluated the impact of the MHC on mental wellness and functioning among youth in Leon, Nicaragua.

**Methods.:**

High school and university students (aged 14–25 years) were assigned to intervention (12-week MHC; *n*  =  567) and control (wait-list; *n*  =  346) groups in a non-randomized design. Both groups completed measures of mental health knowledge, stigma and function at baseline and 12 weeks. Multivariate analyses and repeated measures analyses were used to compare group outcomes.

**Results.:**

At baseline, intervention students showed higher substance use (mean difference [MD]  =  0.24) and lower perceived stress (MD = −1.36) than controls (*p*  <  0.05); there were no other group differences in function. At 12 weeks, controlling for baseline differences, intervention students reported significantly higher mental health knowledge (MD  =  1.75), lower stigma (MD  =  1.78), more adaptive coping (MD  =  0.82), better lifestyle choices (MD  =  0.06) and lower perceived stress (MD = −1.63) (*p*  <  0.05) than controls. The clinical significance as measured by effect sizes was moderate for mental health knowledge, small to moderate for stigma and modest for the other variables. Substance use also decreased among intervention students to similar levels as controls (MD  =  0.03) (*p* > 0.05).

**Conclusions.:**

This pilot investigation demonstrates the benefits of the MHC in a low-and-middle-income youth population. The findings replicate results found in Canadian student populations and support its cross-cultural applicability.

## Introduction

Mental disorders (mental illnesses and addictions) tend to have onset in childhood and adolescence (most frequently in the 15–24 age range) and if left untreated, often carry into adulthood (Kessler *et al.* 2009). Worldwide, it is estimated that 10–20% of youth (<25 years) experience a mental illness or addiction (World Health Organization [WHO], 2016) and impairment of education, interpersonal relationships, physical health and overall quality of life is common in this population (Kessler *et al.* 1995).

Adverse social determinants of mental health include poverty, violence, discrimination, unsafe environments and exposure to manmade and natural disasters (United Nations Population Fund [UNFPA], 2014). About 80% of those with mental illness, addictions or suicidal behaviour live in low- and middle-income countries (LMICs), where such stressors are endemic (WHO, 2009) and 50% of the population is under age 24 (UNFPA, 2014). Despite the burden of mental disorders in these regions (accounting for 21% of the total burden of disease and up to 21% of years lived with disability), health service responses are often limited or inadequate, with stigma, social discrimination and lack of knowledge or misinformation serving as deterrents to help-seeking, and lack of affordability reducing access to care (Gulliver *et al.* 2010; Rodríguez, 2010).

It is well documented that mental health knowledge is deficient worldwide, but particularly so in LMICs (Ganasen *et al.* 2008; Jorm *et al*. 2012), and stigma is common even among mental health professionals in developing regions (Ndetei *et al.* 2009; Roberts, 2010). Improving mental health literacy is an effective way to reduce stigma and discrimination, and enhance positive health outcomes (WHO, 2015; United Nations Economic Scientific and Cultural Organization, n.d. ). Schools are ideal settings for promoting mental health awareness and providing support for young people, and as access points for additional services (Joint Consortium for School Health, 2009; University of California Los Angeles Center for Mental Health in Schools, 2009; Wei & Kutcher, 2012). Life skills training, physical fitness programmes, and peer support groups have been used effectively in some school-based initiatives to improve general well-being (Bonhauser *et al.* 2005; Kumakech *et al.* 2009; Srikala & Kumar, 2010), but specific benefits for mental health were either not fully evaluated or found to be only modest (Barry *et al.* 2013). Reviews of school-based programs have noted that structured initiatives that focus specifically on mental health literacy may have more robust benefits in improving knowledge, coping skills and resilience, increasing help-seeking, and reducing stigma among youth (Weare & Nind, 2011; Fazel *et al.* 2014; Fernandez *et al.* 2016). Better coping is associated with decreased substance abuse (Griffin *et al.* 2002), which is a common maladaptive coping strategy among youth to cope with mental illness and stress (Roberts *et al.* 2009). The efficacy of such programs has been noted to be enhanced by teacher delivery and integration into the regular curriculum (Neil & Christensen, 2009; Weare & Nind, 2011). Recently, various mental health literacy programs led by teachers have been implemented in several countries, resulting in improved mental health knowledge and help-seeking intentions and reduced stigma among students (Skre *et al.* 2013; Kutcher *et al.* 2015*b*; Ojio *et al.* 2015).

In LMICs, human and financial resources are limited, mental health awareness is low and mental health policies are frequently outdated (WHO, 2009). Addressing youth mental health is often a particular challenge. School-based mental health literacy programs may provide at least a partial solution, although their applicability in these regions requires confirmation. One such initiative, a mental health curriculum (MHC), was developed by a Canadian team of mental health experts specifically to target the critical 15–24 year age group of transitional youth (Kutcher, 2015). It includes mental health literacy education for teachers and students, a curriculum guide for teachers, an informational website to support the curriculum, and message board forums for teacher–teacher, student–student and student–teacher discussions. The previous implementation of this program among Canadian students was associated with sustained increases in self-reported mental health literacy and help-seeking intentions, and reductions in negative attitudes toward mental illness (stigma) (McLuckie *et al.* 2014; Kutcher *et al.* 2015*b*; Milin *et al.* 2016). It was also associated with increased mental health literacy and skills among teachers (Kutcher & Wei, 2013; Kutcher *et al.* 2015*a*, 2016). The curriculum was also found to be sustainable, with low cost and time requirements and effective employment of the ‘train the trainer’ model. Furthermore, there is preliminary data from Africa that indicates good cross-cultural applicability, though thus far, only results from teacher training have been reported (Kutcher *et al.* 2015*a*, 2016).

Nicaragua is one of the poorest countries in Central America and the second poorest country in the Western hemisphere (Central Intelligence Agency [CIA], 2016). About 30% of the population lives below the poverty line, i.e. on <$2 CAD per day (World Bank, 2016). Mental disorders are highly prevalent in the local population, and the highest rates of mental illness, addictions and suicide are among young people aged 15–24 years (Caldera Aburto, 2004; Rodriguez *et al.* 2006; United Nations Office on Drugs and Crime [UNODC], 2010; Quinlan-Davidson *et al.* 2014). Due to limited mental health infrastructure, budgeting (mental health is allocated 1% of the health budget, which is itself only 8% of GDP), and expertise (estimated figures of 0.64 psychiatrists and 0.45 psychiatric nurses available per 100 000), upwards of 60% of the population has no access to mental health care (Jacob *et al.* 2007). Mental health services specifically targeted towards transitional youth are sparse, as are the programs to address youth mental health literacy. Often, poor mental health knowledge, reduced access to care, and the stigma of mental illness contribute to reduced help-seeking and delayed clinical intervention (Obando Medina *et al.* 2014). Thus, there is substantial opportunity to implement novel, evidence-based interventions to try to enhance health and mental health outcomes in this high-risk group.

In 2014, a collaborative project between the Department of Psychiatry at the University of Toronto and the Universidad National Autonoma de Nicaragua, Leon (UNAN-Leon) was launched in Leon, the second largest city in Nicaragua. The aim of this pilot investigation was to evaluate the impact of the MHC on mental health knowledge, stigma, and functioning among youth in Leon, Nicaragua. It was anticipated that students who received the intervention would show better mental health knowledge, lower stigma, and improved functioning compared to students who did not.

## Methods

### Study design

This parallel-group, non-randomized study began in December 2013 and ended in June 2016. This time period encompassed several months of project set-up, cultural and linguistic adaptation of components, and website and database development, followed by teacher training, curriculum delivery, baseline and follow-up data collection for teachers and students (follow-up data on teachers was collected for up to 18 months), and subsequently, data analysis and local and regional knowledge dissemination events. Timelines also accommodated the local academic system, which is closed from November to February of each year for the end of academic year holidays and also periodically during the remaining months for religious and national holidays, precluding any project activities from taking place locally during these times. Time allowance for additional school closures due to environmental events, such as earthquakes and hurricanes, which are common in Nicaragua and which did occur during the project period, were also factored into the timelines.

In order to reach all available students in the target age group (15–24 years), all four high schools in Leon (Instituto Modesto Armijo, Instituto Mariano Barreto, Instituto John F. Kennedy and Instituto Nacional De Ocidente) and the local university (UNAN-Leon) were invited to participate in the project. As students in the target age range were mostly found in Grades 9–10 in high school and the first 2 years of university in Nicaragua, participants for the project were drawn only from these grades/years.

Two high schools and several university departments were assigned to the intervention group that would receive the MHC immediately, and the other two high schools and remaining university departments formed the control group that would receive the MHC 1 year later (at the end of the data collection period). Since the participating academic centres were all reluctant to be in the control group, the initial plan had been to assign them to intervention or control based on the timing of agreement to participate. However, the centres all made their decisions within the same short time period, and the local principal investigator (A.H.) decided to allocate them to achieve an equitable balance of intervention *v.* control groups based on school/university department academic reputation (i.e. higher or lower academic record), geographic location (i.e. city centre *v.* city outskirts) and social determinants (i.e. average socio-economic status of students). This also served to reduce selection bias.

To evaluate the impact of the MHC, students in both the intervention and control groups completed self-report questionnaires at baseline (pre-intervention) and 12 weeks (post-intervention). These were administered by the teachers who delivered the curriculum or led the control group classes, assisted by local project team members. The questionnaires surveyed students’ mental health knowledge and stigma, psychological distress, substance use, stress, resilience, and quality of life.

A collaborative, joint decision-making approach was utilized for all implementation decisions and involved both the Canadian and Nicaraguan team members, to maintain accommodation of and relevance to the local context.

### Participants

This was a pilot study involving an educational intervention with a non-clinical population and the target population was students from the high schools and the local university in Leon. As no previous data with this population were available to make *a priori* estimations, no sample size calculation was performed. Convenience sampling was thus used to recruit all students in the targeted high school grades or university years. The sample size was determined pragmatically as all students from the participating institutions who met eligibility criteria for the study were enrolled.

Students were recruited from Grade 9 in high school (so they could be tracked if necessary into Grade 10, the last grade in high school) and from the second year in university (as Year 1 has common coursework for all students and differentiation by the department only occurs in Year 2). All students in the relevant grade/university year were eligible to participate. No students had any impairments that would have impeded their ability to participate in the curriculum.

Since the high schools assigned to the intervention group had a higher number of students in the targeted grade, the sample size of the intervention group as a whole was larger (*n*  =  567) than that of the control group (*n*  =  346).

### Intervention

The intervention group received the MHC (Kutcher, 2015), which consists of six modules considered important to student mental health literacy: (a) stigma of mental illness; (b) understanding mental health and mental illness; (c) information on specific mental illnesses; (d) adolescents’ experiences of mental illness; (e) strategies to address stigma and promote help-seeking; and (f) the importance of positive mental health. An accompanying website provides additional information on mental health concepts through articles, presentations, and videos and stories about youth mental illness and addictions.

For schools and university departments assigned to the intervention group of the study, teachers were trained in curriculum delivery during a 3-day face-to-face teacher training program by the Canadian project team, which was supported by local staff from the Centre for Demographic and Health Research (CIDS) at UNAN-Leon. The teachers were also trained to train other teachers in their schools in MHC delivery, to build a resource base of trained personnel who could continue MHC implementation in the future. Fidelity to curriculum delivery training and to the train-the-train approach were verified through observational ratings by the local project team of randomly selected teaching and training sessions.

The full curriculum was delivered to intervention group students through 1-h, weekly sessions over 12 consecutive weeks. The curriculum and website were only accessible by intervention group teachers and students during the active data collection period. The frequency and duration of website visits by students were tracked.

The control group received no mental health literacy information during the same 12 weeks and did not have access to the curriculum or the website.

### Cultural and linguistic adaptation

The content of the MHC modules, the training material, and the website were translated into Spanish and culturally adapted to fit the Nicaraguan context using local mental health experts, including clinical staff (i.e. psychiatrists and psychologists) from UNAN-Leon and from community mental health agencies. Local partners from UNAN-Leon also led the cultural adaptation and provided the final approvals. As the local students and teachers were mostly familiar with the fundamental mental health issues, the content was highly adherent to the Canadian version of the MHC. Cultural adaptations related largely to use of local colloquial terms and phrases to explain concepts (e.g. ‘attaque de ron’, or ‘attack of the rum’, to explain pancreatitis that can result from prolonged alcohol abuse); descriptive and geographic changes to situational examples to fit the Nicaragua context (e.g. Jane from Canada who was stressed because she lost her job became Maria from Nicaragua whose stress was related to financial needs); and identification of local mental health agencies and services as resources.

For data collection, Spanish versions of available measures were used, and where none were available, measures were translated into Spanish by bilingual (Spanish/English) clinical staff from the local project team at UNAN-Leon and back-translated into English to ensure consistency. Almost all the measures were already available in Spanish. Therefore, the only ones that needed translation were the Mental Health Knowledge and Attitudes Scale (MHKAS) (Kutcher & Wei, 2013) and the General Help-Seeking Questionnaire (GHSQ; Wilson *et al.* 2005).

### Primary and secondary outcomes

The primary outcome was the change in scores between baseline and 12 weeks on the:

- Mental Health Knowledge and Attitudes Scale (MHKAS) (Kutcher & Wei, 2013) from baseline to 12 weeks. The MHKAS evaluates mental health knowledge and stigma. It has two subscales. The Knowledge subscale assesses general mental health knowledge, corresponding to the material contained in the six modules of The Guide. Scores range from 0 to 28 and higher scores indicate better mental health knowledge. The Attitudes subscale examines attitudes related to mental disorders or mental illness. Scores range from 8 to 56 and higher scores indicate better attitudes (i.e. lower stigma).

The secondary outcomes were the changes in scores between baseline and 12 weeks on the following measures:

- The Brief COPE (Carver, 1997) assesses effective and ineffective coping strategies. The effective strategies items range in score from 16 to 64 and higher scores indicate more adaptive coping. The ineffective strategies items range in score from 12 to 48 and higher scores indicate more maladaptive coping.
- The CRAFFT (Knight *et al.* 2002) measures adolescent alcohol and drug use. The acronym ‘CRAFFT’ is built on key words in the included items, e.g. driving in a Car while drunk, using drugs to Relax, etc. Scores range from 0 to 6 and higher scores indicate more problematic substance abuse.
- The General Health Questionnaire – 12 (GHQ-12; Banks, 1983) measures psychological distress due to depression and anxiety. Scores range from 0 to 36 with higher scores indicating more psychological distress.
- The GHSQ (Wilson *et al.* 2005) assesses help-seeking behaviours and intentions. Scores range from 20 to 140 with higher scores indicating better help-seeking tendencies.
- The Health-Promoting Lifestyle Profile II (HPLP II; Walker *et al.* 1987) measures the frequency of self-initiated health behaviours. Scoring is based on the average of the sum of all items; therefore, scores range from 1 to 4 with higher scores indicating better healthy lifestyle choices.
- The Perceived Stress Scale (PSS; Cohen *et al.* 1983) measures perception of stress. Scores range from 0 to 56 with higher scores indicating more perceived stress.
- The Quality of Life Scale (QOL; Burckhardt *et al.* 1989) assesses satisfaction with personal, interpersonal and academic/occupational life domains. Scores range from 16 to 112 and higher scores indicate better quality of life.
- The Resilience Scale – Short Form (RS-14; Wagnild & Young, 1993) measures psychological resilience to stress. Scores range from 14 to 98 and higher scores indicate better resilience.

All the measures have an emerging or well-established psychometric and cross-cultural adequacy in youth populations.

### Data analysis

Descriptive analyses and Chi-Squares of similarities and differences in demographics between the two groups were conducted. Multivariate analyses of covariance (MANCOVA) were used to compare the groups on baseline scores and repeated measures MANCOVA were conducted to compare pre-post changes in scores between the groups over time while controlling for any demographic differences.

### Ethical considerations

Ethics approval was obtained from the Ministry of Education of the province of Leon, Nicaragua for the participation of the selected academic institutions. In addition, consistent with local standards, the local school boards and UNAN-Leon reviewed the protocol and provided consent for teacher and student participation in the curriculum and for administration of questionnaires. Individual students could choose not to complete measures without consequences.

To ensure confidentiality, anonymity, and security of the data, all participating students were assigned unique, anonymized identifiers at enrolment to the study. These identifiers were used for data entry and to track student responses over time; no personal identifying information was collected. The data were stored behind double locks, on computers or filing cabinets to which only project personnel had access.

## Results

While teacher-specific data are discussed in more detail in a manuscript currently in preparation, in brief, intervention teachers showed good fidelity to training, achieving over 85% fidelity in curriculum delivery as determined by project team observational ratings.

**Fig. 1.**
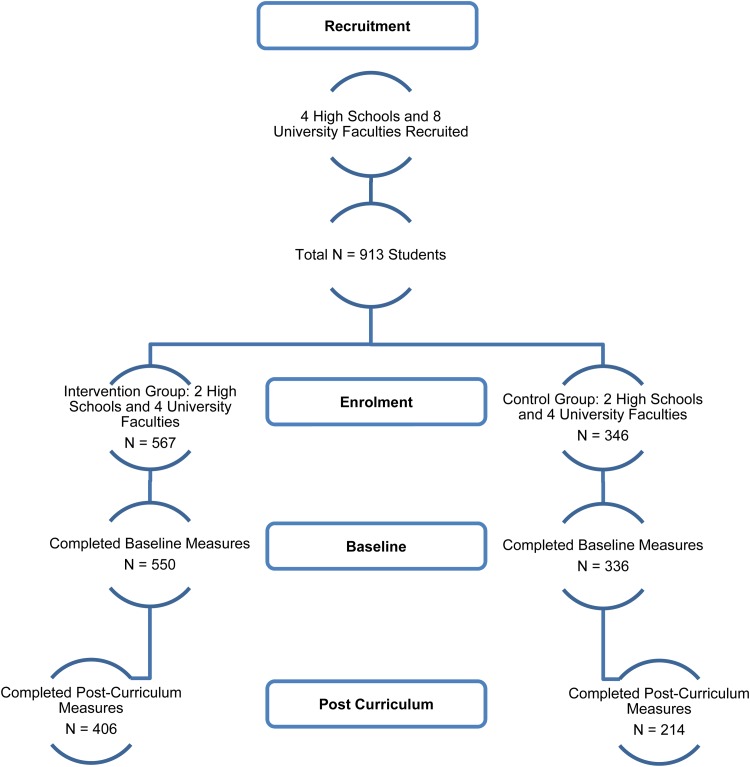
CONSORT Diagram.

A total of 913 students from the four high schools and university departments were recruited (intervention group  =  567, control group  =  346). A total of 886 participants completed baseline measures (intervention group  =  550, control group  =  336). A total of 620 students (intervention group  =  406, control group  =  214) completed measures at 12 weeks (post-intervention) (see Fig. 1).

### Pre-intervention (baseline)

Across the sample, 61% was female and 28% had a low family income, defined as <$225 CAD per month. About 19% lived at or below the poverty line (family income of <$110 CAD per month). The age range was 14–25 years, of which 58% were in the 17–19 year age group. Thirteen percent (13%) reported receiving mental health treatment in the past year, and 34% reported abusing substances in the past year. Of those who sought mental health treatment, 57% were female and 66% were aged 17–19 years. Of those who reported using addictive substances, 59% were female and 61% were in the 17–19 year age range.

A comparison of demographic data between the intervention and control groups found that there was a higher proportion of female participants in the control group than in the intervention group (χ^2^(1, *N*  =  912)  =  9.02, *p*  =  0.003). The groups were similar on all other demographic parameters (see Table 1).

**Table 1.** Demographic comparison of the intervention and control groups at baseline

	Intervention (*n* = 567)	Control (*n* = 346)
Mean age (s.d.)	17.62 (2.03)	18.11 (1.96)
Gender (%)		
Female	55.7% (*n* = 316)	65.8% (*n* = 227)
Male	44.3% (*n* = 251)	34.2% (*n* = 118)
Marital status		
Married	2.0%	1.5%
Single	92.8%	93.0%
* *Common Law	4.7%	5.2%
* *Separated/Divorced	0.5%	0.3%
Employment Status		
Part-time	4.3%	4.4%
Not currently working	0.9%	0.0%
Student	94.8%	95.6%
Family income (per month)		
<2500 Cordobas (~ <$110 CAD)	18.5%	19.4%
2501–5000 Cordobas (~ $111–$225 CAD)	28.9%	27.4%
5001–10 000 Cordobas (~$226–$450 CAD)	21.0%	22.3%
>10000 Cordobas (~ $450 CAD)	15.6%	14.4%
Don't Know	16.0%	16.5%

To control for the potential influence of group differences in gender proportions, gender was included as a covariate in the group comparison of scores on pre-intervention measures.

The intervention and control groups scored comparably on most of the self-report measures completed at baseline (*p* > 0.05) (see Table 2). There were significant group differences on the CRAFFT and PSS measures, with intervention group students showing higher levels of substance abuse (*p*  =  0.01) and lower levels of perceived stress (*p*  =  0.01) than control group students, though with very weak effect sizes.

**Table 2.** Comparison of mean baseline scores on self-report measures between the intervention and control groups, using MANCOVA

	Intervention group (*n* = 550) Mean (s.d.)	Control group (*n* = 336) Mean (s.d.)	Mean difference (Intervention – Control)	F	p*	95% CI	Partial η^2^
Brief COPE – Adaptive	24.83 (6.71)	25.15 (6.48)	−0.34	0.55	0.46	(−1.25 to 0.57)	
Brief COPE – Maladaptive	17.89 (4.52)	18.24 (4.37)	−0.36	1.37	0.24	(−0.97 to 0.25)	
CRAFFT	1.12 (1.38)	0.89 (1.41)	0.24	6.21	0.01*	(0.05 to 0.43)	0.01
GHQ-12	14.88 (4.97)	15.18 (5.61)	−0.31	0.72	0.40	(−1.02 to 0.41)	
GHSQ	30.65 (10.75)	30.99 (10.32)	−0.35	0.22	0.64	(−1.79 to 1.10)	
HPLP II	2.18 (0.42)	2.15 (0.47)	0.04	1.43	0.23	(−0.02 to 0.10)	
MHKAS – Knowledge	12.03 (4.04)	12.43 (3.80)	−0.38	1.88	0.17	(−0.92 to 0.16)	
MHKAS – Attitudes	32.58 (6.29)	32.36 (6.13)	0.33	0.56	0.45	(−0.52 to 1.17)	
PSS	24.60 (7.37)	25.93 (7.42)	−1.36	6.97	0.01*	(−2.37 to −0.35)	0.01
QOL	83.45 (12.76)	83.96 (12.71)	−0.67	0.58	0.45	(−2.41 to 1.07)	
RS14	76.28 (15.29)	74.84 (15.39)	1.37	1.64	0.20	(−0.73 to 3.46)	

*Statistically significant, *p* < 0.05.

### Post-intervention (12 weeks)

Once again, to control for the potential influence of group differences in gender proportions, gender was included as a covariate in the group comparison of changes in scores on the measures over time. The repeated measures nature of the analysis also innately controlled for baseline differences on the CRAFFT and PSS across all outcomes.

Between baseline and 12 weeks, and compared with control group students, intervention group students showed significantly better scores on measures of mental health knowledge and attitudes (stigma), adaptive coping, health-promoting behaviours, and perceived stress (*p*  <  0.05) (see Table 3). The effect sizes were moderate and the confidence intervals were narrower for changes in mental health knowledge, while effect sizes were small to moderate for changes in stigma, and modest for changes in adaptive coping, healthy lifestyle choices and perceived stress, with wider confidence intervals (see Table 3). Of note, substance abuse scores for the intervention group also improved from baseline, resulting in no group differences *v.* the control group (who had reported lower levels of substance abuse at baseline compared with the intervention group) post-intervention (*p*  =  0.69).

**Table 3.** Comparison of group differences in changes on self-report measures from pre-curriculum to post-curriculum, using repeated measures MANCOVA

	Intervention Group (*n* = 406)		Control Group (*n* = 214)		Mean difference (Intervention– Control)	F	p*	95% CI	Partial η^2^
	Pre Mean (s.d.)	Post Mean (s.d.)	Pre Mean (s.d.)	Post Mean (s.d.)					
Brief COPE – Adaptive	24.97 (6.85)	25.57 (6.65)	25.50 (6.40)	23.42 (5.96)	0.82	4.41	0.04*	(0.05 to 1.59)	0.01
Brief COPE – Maladaptive	17.83 (4.55)	18.46 (4.36)	18.37 (4.43)	17.78 (4.17)	0.07	0.08	0.78	(−0.44 to 0.58)	
CRAFFT	1.17 (1.42)	0.92 (1.32)	1.07 (1.54)	0.93 (1.36)	0.03	0.16	0.69	(−0.13 to 0.20)	
GHQ-12	14.74 (4.94)	15.58 (4.81)	15.51 (5.70)	15.45 (4.41)	−0.33	1.23	0.27	(−0.91 to 0.25)	
GHSQ	30.98 (10.39)	35.77 (9.78)	31.41 (10.26)	33.60 (9.75)	0.86	1.59	0.21	(−0.48 to 2.20)	
HPLP II	2.19 (0.41)	2.31 (0.46)	2.17 (0.47)	2.21 (0.49)	0.06	4.33	0.04*	(0.003 to 0.11)	0.01
MHKAS – Knowledge	12.15 (3.99)	14.38 (4.72)	12.21 (3.94)	10.78 (4.55)	1.75	46.25	<0.0005*	(1.25 to 2.26)	0.07
MHKAS – Attitudes	32.47 (6.32)	36.84 (6.51)	32.89 (6.12)	32.93 (5.40)	1.78	25.01	<0.0005*	(1.08 to 2.48)	0.04
PSS	24.53 (7.19)	22.27 (7.50)	26.38 (7.49)	23.80 (7.05)	−1.63	13.70	<0.0005*	(−2.49 to −0.76)	0.02
QOL	83.56 (12.79)	83.78 (13.90)	83.90 (12.31)	83.64 (12.56)	−0.11	0.02	0.88	(−1.61 to 1.38)	
RS-14	76.97 (15.13)	77.12 (15.18)	73.95 (16.61)	76.70 (14.56)	1.75	3.52	0.06	(−0.08 to 3.58)	

*Statistically significant, *p* < 0.05.

There were no significant group differences on any of the other measures, i.e. psychological distress, maladaptive coping, help-seeking tendencies, resilience or quality of life.

The rate of attrition from the study between baseline and post-intervention differed significantly between groups, with lower attrition found in the intervention group (intervention group 26.2%, control group 36.3%) (χ^2^(1, *N*  =  886)  =  10.18, *p*  =  .001).

Website data indicated that 100% of intervention group students (*n*  =  567) visited the curriculum website at least once and 300 (52.9%) made repeated visits.

## Discussion

While the MHC has been implemented in other LMICs, notably Malawi and Tanzania, published data have so far focused mainly on the impact of the MHC on teachers from those regions (Kutcher *et al.* 2015*a*, 2016). This is the first study to report on outcomes in a non-Canadian student population from a low-income country. The results confirmed that the MHC significantly improved mental health knowledge and reduced stigma among youth in Leon, Nicaragua who received the curriculum. Our findings also document the positive impact of the MHC in several other key life domains – coping strategies, perception of stress, healthy lifestyle choices, and substance abuse practices. It is acknowledged that the investigations had several limitations, which are reviewed later in the discussion.

The positive impact of the MHC on mental health knowledge and stigma in the Nicaraguan student intervention group is consistent with previous findings with Canadian students (McLuckie *et al.* 2014; Kutcher *et al.* 2015*b*; Milin *et al.* 2016). Notably, the most recent study (McLuckie *et al.* 2014) was an RCT that utilized a similar design (i.e., MHC added to usual teaching *v.* the latter alone) to the current project. While baseline scores for attitudes towards mental illness in our sample were comparable with those found with Canadian students prior to intervention, scores for mental health knowledge were lower (McLuckie *et al.* 2014; Kutcher *et al.* 2015*b*); this variance is likely due to the poorer basic mental health literacy in LMICs (Ganasen *et al.* 2008; Jorm, 2012). However, the strength of the positive change in mental health knowledge and stigma following the intervention in our sample were in the same moderate to large range as those reported with Canadian students (McLuckie *et al.* 2014; Kutcher *et al.* 2015*b*). These findings indicate that the MHC was effective in improving mental health knowledge and reducing stigma among students in Nicaragua despite poor understanding of mental health issues at baseline.

As anticipated, the intervention group also showed significant improvement in adaptive coping, healthy lifestyle choices (nutrition, exercise, socializing, stress management) and perceived stress compared with controls. Substance abuse scores for the intervention group, which was higher than for the control group at baseline, became comparable with the control group post-intervention, indicating that the MHC had a positive impact on adaptive behaviours. Previous studies with youth have also noted associations between better coping and lower perceived stress (Burger & Samuel, 2017), lower substance use (Brady *et al.* 2009; Chua *et al.* 2015) and better lifestyle choices (Chua *et al.* 2015; Kurspahić-Mujčić *et al.* 2014). However, this range of functioning was not assessed in previous Canadian studies with the MHC (McLuckie *et al.* 2014; Kutcher *et al.* 2015*b*; Milin *et al.* 2016) and thus our findings add to the literature on its benefits.

At baseline, both the intervention and control groups exhibited a high degree of perceived stress, but the level was significantly lower in the former compared with controls. The intervention group also showed higher substance abuse scores at baseline. These findings may seem counterintuitive but are explainable as it is well known that substances are frequently used by youth to self-medicate to alleviate psychological distress (Debnam *et al.* 2016; Nair *et al.* 2016), often because of the misbelief that substance use can help to reduce stress (Canadian Centre on Substance Abuse [CCSA], 2007; Roberts *et al.* 2009). However, in reality, substance abuse provides only a temporary, if any, reduction of stress and actually exacerbates it (CCSA, 2007), and this may be reflected in the continued high levels of perceived stress in the intervention group at baseline despite their higher substance use. It is therefore noteworthy that decreases in substance abuse in the intervention group post-intervention were accompanied by increased adaptive coping and reduced perceived stress. It is possible that intervention students were applying better stress management techniques drawn from the MHC, that offset the misuse of substances as a coping mechanism.

The intervention and control groups both reported similar and acceptable levels of resilience and quality of life at baseline and post-intervention. While this may seem contrary to expectations, it must be highlighted that in Nicaragua, poverty often forces children into the workforce at an early age, despite free education (World Bank, 2011; CIA, 2016; UNIDOS Nicaragua, 2016). While 87% of children enter the school system, only 40% enter high school and only 16% graduate. Thus, our student sample, recruited from the final years of high school and early years of university, may be more intellectually gifted or alternatively, may represent a more privileged segment of the youth population. It has been noted that even families with low incomes often strive to support their students, resulting in better quality of life and more resilience.

This study endeavoured to improve methodological rigour compared to previous investigations with the MHC. Most prior studies utilized open trial designs without control groups (McLuckie *et al.* 2014; Kutcher *et al.* 2015*b*), which limited interpretation of results; the use of a parallel-group design thus contributed to a more valid demonstration of the benefits of the MHC. Fidelity to curriculum delivery and assessment was ensured by having the local project team observe and rate randomly selected teaching sessions and assist also with the administration of assessments (this is further elaborated on in a teacher-specific manuscript currently in preparation). The cultural and linguistic adaption of the MHC, and involvement of local project staff in evaluating, validating and approving it, enhanced its cross-cultural fit. Attrition rates from the project over the course of MHC implementation were significantly higher in the control group than in the intervention group – this despite the fact that absenteeism is a significant academic issue at all levels of education in Nicaragua (World Bank, 2011) – indicating that the content of the MHC was of sufficient interest to intervention group students that they maintained better attendance.

A key finding of this investigation is that enhancement of mental health literacy and function in youth can be achieved with the use of existing resources at low cost. Rather than being delivered by special trainers or requiring extra school time for implementation, the MHC was delivered by usual classroom teachers and embedded within the usual school curriculum. Several previous publications have confirmed the effectiveness of this strategy in high-income countries (Skre *et al.* 2013; Kutcher *et al.* 2015*b*; Ojio *et al.* 2015), and this investigation validates its applicability to low-income settings with limited mental health resources.

The broader impact of the MHC on student function was particularly significant as the sparse youth mental health services in Nicaragua are not able to meet the need demonstrated by the high rates of youth psychiatric and addiction morbidity (UNODC, 2010; Quinlan-Davidson *et al.* 2014; WHO, 2015). Increased awareness of mental health issues tends to lead to increased demand on services, which is often a challenge for low-income countries due to financial and human resource restrictions. Alternate methods of service delivery that can be accommodated within local fiscal and expertise restrictions are thus urgently needed. The MHC may provide one such option. By improving student function across several domains over a relatively short time period and by utilizing current school resources, the MHC demonstrated its versatility as a vehicle both for mental health education and mental health intervention. If these benefits are replicated in further research, the MHC may be a useful tool for enhancing both school system capacity and mental health infrastructure, with little additional cost and without interruption of existing school practices. Current findings also demonstrated that the education system in Nicaragua can play the important role of filling some part of the mental health gap, as hubs of mental health knowledge and support for youth. Further, as teachers were trained both to deliver the MHC and to train others in turn, and as curriculum material (teaching material, website content) have remained accessible to the participating schools and have been made shareable with other local academic institutions post-project, the MHC is a new and valuable resource for the local and wider community, that costs little to implement, and is highly sustainable.

Our study had several limitations. The study was unblinded, which may have contributed to expectancy effects and affected the self-reported changes in stigma and functioning in the intervention group. It was also non-randomized, as an assignment to the intervention or control group was based on the local team's judgement regarding equitable assignment based on school and student characteristics. It is thus possible that a factor common to the intervention group, rather than the MHC, may have contributed to observed improvements. Against the presence of this bias are the observations that the groups were similar both demographically and on baseline psychosocial functioning (other than on the PSS and CRAFFT) and that improvements were noted in the intervention group even after controlling for baseline differences. Among other limitations, data collection was only short-term and thus, sustainability of improvements long-term is unknown. Although the project focused on specific school grades/years, our student sample had a wide age range (14–25 years), which may have led to a variance in ability to understand and apply MHC concepts, and thereby reduced group differences. Furthermore, Leon is a small city and since the MHC generated a lot of local interest, the possibility of information sharing between the two student groups cannot be ruled out, which may have reduced any group differences post-intervention, as well. Finally, only a minority of students enter high school and university, and as such, caution must be used in extrapolating these findings to the broader youth population of Nicaragua.

In conclusion, the school-based MHC program was effective in improving mental health knowledge, reducing stigma and enhancing function among youth in Leon, Nicaragua. These results replicate previous findings in Canadian student samples and confirm the cross-cultural applicability of the MHC. This pilot investigation suggests that the MHC may be an effective, low-cost option to improve mental health literacy and adaptive function. It also demonstrates the feasibility of collaborative, large-scale intervention research across multiple institutions in low-income countries. Replication of the study through randomized, larger-sample trials would be useful for further evidence to support consideration of the MHC for scale-up options in LMICs.
